# Sensitivity and quality factor improvement of photonic crystal sensors by geometrical optimization of waveguides and micro-ring resonators combination

**DOI:** 10.1038/s41598-024-52363-2

**Published:** 2024-01-23

**Authors:** Vahid Fallahi, Zoheir Kordrostami, Mehdi Hosseini

**Affiliations:** 1 Department of Electrical and Electronic Engineering, and Research Center for Design and Fabrication of Advanced Electronic Devices, Shiraz University of Technology, Shiraz, Iran; 2 Physics Department, Shiraz University of Technology, Shiraz, Iran

**Keywords:** Engineering, Mathematics and computing, Nanoscience and technology, Optics and photonics, Physics

## Abstract

In this work, the process of designing and simulating optical sensors based on photonic crystal (PC) micro-ring resonators (MRRs) has been investigated. According to the PC type, different waveguides and resonators can be designed, and various topologies can be proposed from their combination, for optical sensor applications. Here, the investigated MRR is of the symmetrical micro-hexagonal ring resonator (MHRR) type. Different arrays of MHRR arrangement have been designed to investigate their effects on the output spectrum. The results of the design and simulation of different topologies have been analyzed and compared with other numerical researches. Considering all the necessary aspects of PC optical sensors, a detailed and comprehensive algorithm has been presented for designing these devices and choosing the optimal structure. In a more complementary process, the effects of reflector rods have been investigated, which indicates the existence of similarity and compatibility in the design between the distance of reflector rods and the length of MHRRs to obtain the optimal structure. Finally, the effect of different values of lattice constant and radius of dielectric rods on FWHM, transmission (TR) and resonant wavelength is studied, and the most optimal mode is presented. In order to measure the performance of the proposed optimal sensor, its application for gas detection has been analyzed. TR, FWHM, quality factor (QF), sensitivity (S) and figure of merit (FOM) of the proposed sensor were equal to 96%, 0.31 nm, 2636, 6451 nm/RIU and 2960 RIU^−1^ respectively. An examination of results from similar research indicates a rational and effective approach for generating diverse topologies, aiming to attain the most optimal configuration for optical sensors employing MRRs. Furthermore, employing a systematic design process based on established principles and the proposed algorithm helps prevent arbitrary parameter variations, facilitating the attainment of desired outcomes in a more streamlined and efficient manner. Given the comprehensive nature of this research, it presents a viable solution for designing optical devices based on MRRs for use in optical integrated circuits (OICs) applications.

## Introduction

With the advancement of technology and the introduction of optical integrated circuits (OICs), the use of micro-ring resonator (MRR) devices has become one of the most important and integral elements of this field^1–3^. MRRs are devices created by bending waveguides in a closed loop^4^. Due to their nature, these optical devices can be used in different substrates. The small size of a few micrometers and using novel geometries and topologies have provided various applications with interesting characteristics and results^3–5^. Among the numerous applications of these optical devices, we can mention their application in the field of lasers^6^, nonlinear optics^7^, optical sensors and biosensors^1,8^, quantum optics^9^, optical logic circuits^10^, opto-mechanics^11^, photothermal^12^ and chemical sensors^13,14^.

Due to the specific characteristics of MRR devices, their use for the optical sensor is much more evident. The emission of light inside the waveguide and its coupling to the MRR will cause whispering gallery mode with resonance wavelength in the output spectrum^15^. These resonant wavelengths and the required conditions can be widely used in the realization of the detection of different optical sensors. The main advantage of MRRs in creating optical sensors compared to Fabry Perot (FP) resonators is that they don't need any mirrors, which is a huge advantage in the discussion of OICs^4^. On the other hand, compared to Mach–Zehnder interferometric (MZI) devices, MRRs are relatively smaller in size and more sensitive, considering the strong confinement of light inside the ring and a stronger interaction between light and the analyte^8^. The detection process in these devices is based on the changes made in the input signal due to the sensing element. The changes also depend on the refractive index of the rings and the overall structure. Finally, by evaluating the optical properties including intensity, phase, polarization, absorption, resonance momentum, the presence or absence of substance, its concentration, volume and type can be understood^16^.

Transmission (TR) and absorption (Ab) coefficient, free Spectron range (FSR) and FWHM play a significant role in the realization of MRR based optical sensors. These parameters generally determine other quantities like quality factor (QF), sensitivity (S), figure of merit (FOM) and limit of detection (LOD), which ultimately determine the final performance of optical sensors. The materials, structural geometry, combination and special arrangement of MRRs can change or adjust the mentioned quantities^17^. Designing physical parameters of MRRs play a significant role on the obtained results. For example, the type of waveguide coupled to MRR and its effective length directly affects the FWHM and QF^15^, also the number and type of arrangement of MRRs change the values of TR and Ab coefficient^4^. In addition, the value of FSR, which determines the distance between two resonant wavelengths, shows noticeable changes with increasing the number of MRRs. For this reason, it is very necessary to examine the arrays of MRRs along with waveguides and the parameters involved in them in order to obtain the optimal device in the sensing process.

**Figure 1:**
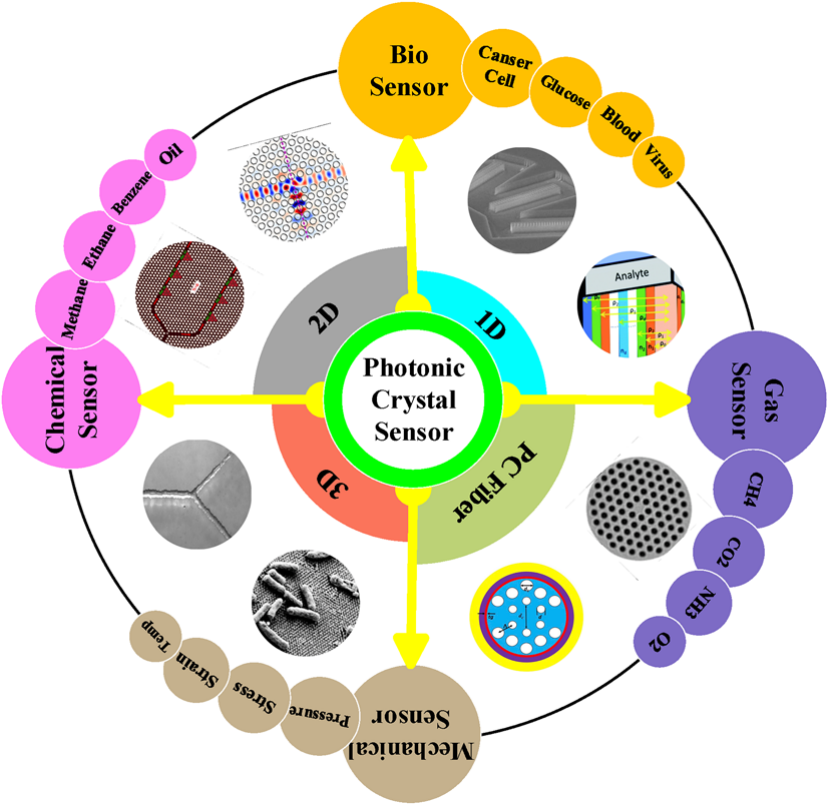
Applications of photonic crystal sensors.

Periodicity in photonic crystals (PCs) causes photonic band gap (PBG)^18^. The presence or absence of periodicity in X, Y and Z directions has created three general structures of 1D, 2D and 3D along with fibers based on PCs^19–21^. Finally, by using these different structures, various sensors have been proposed, which can be divided into four general categories, including biosensors, gas sensors, mechanical sensors, and chemical sensors, each of which is also divided into different subsections as shown in Fig. 1^22–29^. Among the presented MRRs based on PCs, X-shaped, circular, hexagonal, square, rectangular, etc., are widely used in the structures of optical sensors^30^. In addition, these waveguides can also have different variations according to coupling with MRR, which increases the variety in the design of optical sensors based on PCs. It is important to apply theoretical relationships in the field of topology and arrays of MRRs based on PCs. It should be noted that miniaturization into the nanoscale, as well as the quantum states in these structures, can add new features^31^.

As mentioned, using different arrays and topologies of MRRs based on PCs, as well as different waveguides, it is possible to provide sensors with a great variety^32^. In this regard, various paper have been presented by researchers in different ways mentioned above. Fallahi et al.^33^, using a MRR with middle channel waveguide, presented an optical sensor for detection of alcohols, which had high QF and S of 1092 and 745 nm/RIU, respectively. Also, Bahadoran et al.^1^, using double-slot-waveguide based MRR, presented a biosensor with a QF and S above 524 and 248 nm/RIU respectively. Liu et al.^34^, presented a PC topological MRR biosensor for detecting of hemoglobin concentration. In this research the QF and S has been reported 1256 and 798 nm/RIU respectively. Hajshahvaladi et al.^35^, presented a refractive index sensor with a split-ring resonators with hexagonal cavity with QF 2412 and S 1250 nm/RIU. Butt et al.^36^, using MRR based on metal–insulator–metal (MIM) designed a gas sensor with QF 18 and S 1320 nm/RIU. Meanwhile, by examining different topologies, a specific structure can be achieved, and then optimization processes are carried out on its parameters to achieve the best efficiency of the structure. In this work, using a new approach, we intend to examine these processes and their results. Finally, with a comprehensive and complete analysis, check all these cases and provide the most optimal device. In this work, which has been less discussed in this style previously, various structures have been investigated to achieve the optimal structure of a sensor. The innovation lies in the study and comparison of different topologies and arrangements of MRRs along with waveguides, seeking the most suitable design for sensor application. The paper proposes the best structure as the PC sensor and discusses that many of other topologies and arrangements do not lead to a high sensing performance. Investigations are not only focused on the array and topologies, but in a supplementary work, the effects of various parameters in the design, including optical reflector, etc., have been carried out to reach the optimal possible state of the sensor structure. Finally, by presenting a specific algorithm, the principal method of designing structures based on PCs has been presented. For this purpose, in the second part, a general review of optical MRRs in different arrangements and their effects on the sensitivity has been done. In the third part, by presenting a suitable process flow and based on possible combinations of MRRs, the most optimal sensor will be selected. Also, the effects of various parameters involved in the design of this case, including the lattice constant and the radius of the dielectric rods, along with the effects of the reflector rods and the distance of the waveguide coupling region and MRR, have been investigated. In the fourth part, using this optimal structure, an optical sensor has been presented for gas applications.

## Design procedure for optical ring resonator sensor

**Figure 2:**
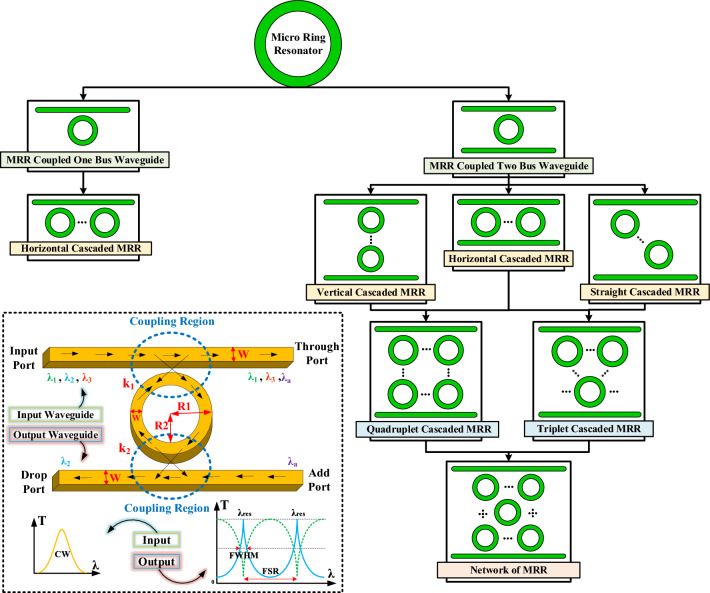
Different classifications of optical MRRs in combination with waveguides.

Optical MRRs are versatile devices that are consist of several waveguides, at least one of which is a closed loop^37^. In these devices, the light enters the structure through a waveguide and is coupled to the MRR. The light propagates inside the waveguide and if the conditions of light intensification and its coupling to MRR are met, due to the periodic boundary conditions that require the repetition of light going back and forth in it, it causes resonance modes to appear in the output spectrum. These resonance modes appear as absorption or transmission peaks according to the MRR structure and its materials. Figure 2, shows the MRR device, which includes input waveguides, output waveguides, ring, coupling region, and its output spectrum is given as a function of the reciprocating phase of the intensified light. The field amplitude enters the waveguide through the input port and after coupling to the MRR which is responsible for resonance, is transferred to the output port. It should be noted that the response of the add/drop port has a resonant transmission peak and the response of the through port appears as a resonant absorption peak. The design parameters involved in MRR structures include coupling length, outer radius (R1), inner radius (R2) and waveguide gap width. By tuning these parameters, the values of cross-coupling amplitude (k) and self-coupling (coupling region) between the access waveguide and MRR, the transmission and the resonance frequency can be controlled. It should be noted that the radius of the ring is considered as an average of inner and outer radii^4^.

The sequence, balance and symmetry in the arrangement of MRRs create different topologies, characteristics and provide special and diverse results and applications. Among these applications, the fields of slow light^38^, optical sensors^8^, optical logic circuits^10^ and also optical telecommunication^39^ could be mentioned. Among the different topologies of these devices, according to their arrangement, vertical, horizontal and diagonal geometries are remarkable. By combining these different positions, other special topologies such as triple, quadruple, and network (or pentagon) topology can be achieved^4,37^. The specific and complete classification of these different topologies, which are unique in their kind, as well as how to create and combine them, is shown in Fig. 2.

In the MRR arrays with different geometries and common waveguides, resonators can be coupled to each other or waveguides, and the output of each MRR is transferred to the next step^37^. In general, the accumulation of light outside the resonance range inside the MRRs is prevented and able to propagate in the arrays^40^. However, the energy transfer along the arrays will be significantly slower because it takes longer for the light in each MRR to transmit to the next loop. The main advantage of devices based on MRRs is the one-way emission of light^41^. The input signal is sequentially coupled to all MRRs, but the presence of a limited loss in each MRR causes light scattering around the resonance range, which in large arrays, the losses become more noticeable and cause significant signal attenuation. The backward propagation of the reflected wave in the MRR devices, makes these topologies more practical as alternating gratings in optical reflectors^42^. In horizontal topologies, it is important to adjust each MRR and precisely control the resonant wavelength. Meanwhile, in vertical topologies, the distance between each ring must be carefully adjusted^4^. Finally, the performance of each array can be optimized by designing and adjusting the involved parameters and the coupling constants.

In network, quadruple and triple topologies, according to the vertical, horizontal and diagonal arrangements of MRR, the light will transmit different paths in the device, which will cause interference in the waves. This process causes the occurrence of null frequencies from transmission or absorption. This prevents configuring arrays containing individual numbers of MRRs^4^. In such configurations, the coupling between anti-emissive modes creates back-reflective modes in the input waveguide, which makes them difficult to use in OICs.

In general, there are no restrictions in the arrangement, design, and application of the topology of different MRRs^16,41,43^. However, one of the most important challenges in this field that should be paid attention to is the mismatch of wavelength optical resonances. For this reason, proper tuning of MRR is necessary to create precise resonance modes, which relies on the development of the manufacturing process and suitable architectures of these devices for OIC applications^8^.

## Design and optimization

In order to investigate the combination of different waveguides with MRR arrays for different topologies, five types of optical waveguides have been considered. The proposed MRR is of symmetrical hexagonal type. The rods are made of silicon in the air substrate and the lattice is triangular with a 460 nm lattice constant (*a*), and the radius of the dielectric rods is equal to 100 nm. In order to maintain simplicity and flexibility, the size of the dielectric rods and the lattice constant of the proposed micro-hexagonal ring resonator (MHRR) are unchanged and equal to the overall structure of the PC. For fabricating the proposed MHRR, it is enough to remove the dielectric rods of the PC to create a closed loop. With a combination of the proposed MHRRs, horizontal single channel waveguide, diagonal double channel waveguide, horizontal double channel waveguide, middle channel waveguide and diagonal channel waveguide with horizontal input could be designed. The proposed base structure investigated in this article is made of 27 dielectric rods in the *x* direction and 21 rods in the *z* direction. The software used is Rsoft Cad-Layout and the number of time steps in the simulations was about 6000 in this software the plane wave expansion (PWE) was used to extract and check the PBG and the finite-difference time-domain (FDTD) method was used to obtain the results of the output spectrum. The input light to the structures is Gaussian type with input power of 1 mW/µm^2^. Also, the investigations carried out in the resonance wavelength range between 810 to 830 nm. The number of dielectric rods, absorption and transmission spectra, FWHM, FSR and resonance wavelength have been investigated. Also, in addition to quantitative investigations, the qualitative analysis of the results obtained from the design and simulation of the presented topologies has been done. It should be noted that by adding more MRRs, the structural size has also changed by increasing the number of dielectric rods in two directions. In this research in addition to add more MRRs, the effects of changing the distance between MRRs in different arrays have also been analyzed. This point is important in order to investigate the different effects of light coupling in terms of the type of waveguide and MRRs arrangement.

The first investigated topology is a single channel waveguide in combination with MHRR which is presented in Table 1. This type is known as the most common optical sensor structure, which is created by removing the add/drop waveguide^32^. In this structure, due to the smallness and simplicity of the design, the simulation time is shorter. The important point in this structure is the multi-peak absorption, which produces problems in some applications, the reason is the weak coupling of the light between the waveguide and the MHRR. The second topology is the combination of the middle channel waveguide with MHRR. This type of topology is suitable for combining and coupling with optical fibers due to the specific type of design. In this topology, due to the direct movement of the wave in the waveguide and the coupling of the MHRR, wider waves are able to pass. The third topology is created by combining two input and output channel waveguides and placing the proposed MHRR in the middle of them. This design is the most promising sensor structure for various applications^8,32^. The fourth topology is proposed from the combination of horizontal channel waveguide as input and oblique channel waveguide as output. Also, in the fifth structure, the output channel waveguide is oblique. These five structures are proposed to cover almost all possible cases of combining waveguides with MHRR. Considering the results of the design and simulations, it can be considered practical to use arrays with two channel waveguides because they enable a higher transmission along with a narrower FWHM. The reason can be attributed to the limited reflection of light to their input waveguide, and the output waveguide in the structure prevents the backward transmission of light in them. Also, due to the lack of direct wave movement in these topologies, the transmitted wavelengths are separated from the central wavelength and are filtered, which can cause a narrower wave to pass through.

**Table 1:** Design and study of various PC sensors using the one MHRR and channel waveguides.

Case	Schematic design	Special description (⁕), feature (⁘) and drawbacks (⁜)	Design	Results			
			Rods (X × Z)	Simulation	λ (nm)	FWHM (nm)	TR (%)
**1**		**⁘**Absorption has been substituted for Transmission **⁘** High absorption **⁘** Simple design **⁘** Short simulation time	27 × 21		819.2	1	100
**2**		**⁘** Simple design **⁘** High transmission **⁘** Short simulation time **⁜** Wide FWHM	27 × 21		818.2	2	95.2
**3**		**⁘**Narrow FWHM **⁘** High transmission **⁘ **Simple design **⁘** Short simulation time	27 × 21		819.8	0.31	96.8
**4**		**⁘** High transmission **⁘** Simple design **⁘** Short simulation time	27 × 21		817.9	0.5	93.2
**5**		**⁘** High transmission – Simple design – Short simulation time	27 × 21		818.3	0.6	99

According to Table 1, the presence of different arrangements of MRRs in the final results has various effects on device performance. For improving the device performance, the arrangement of MHRR arrays and topologies with the presence of proposed waveguides have been investigated in the following studies. It should be noted that, given that all topologies have similar parameters, such as the radius and type of the dielectric rods and the substrate in the structures, the sensitivity of these structures falls within the range of 820 nm/RIU to 880 nm/RIU. According to Table 2, by adding the second MHRR to the sensor structure with a single channel waveguide, the resonance wavelength is still multi-peaks. The important point is that with the increase of the distance between MHRRs, the all-resonance peaks approach to each other and eventually merge into a single broad peak. Therefore, it can be stated that increasing the distance between MHRRs has caused a more precise control of the resonant wavelength, which is in agreement with the content expressed in the theoretical section. But this process decreases the absorption, which leads to an increase in optical losses. On the other hand, increasing the size of the structure will increase the simulation time and the complexity of optimizing the structure. In the next study, the same process is repeated for the sensor with the middle channel waveguide. Adding the second MHRR has reduced the FWHM, which has improved the performance of the optical sensor. Furthermore, increasing the distance of MHRRs has caused the widening of the resonance. Adding second MHRR here caused a more precise adjustment of the resonant wavelength. But it has resulted in a decrease in the amount of transmission and also led to the broadening of the resonance. Increasing the distance between horizontal MHRRs has also followed a similar process as in the previous two cases. The presence of dual channel waveguides in the optical sensor structure provides the use of vertical and diagonal MHRR arrangements. For this purpose, in the continuation of the investigation process, the array of proposed MHRRs is placed vertically next to each other. The results of this case indicate the narrowing of FWHM, but multi-peak output has caused the inefficiency of this topology. The diagonal arrangement of the MHRR array also had the same performance even with the change of arrangement type direction. The triple array of MHRRs is presented in Table 2 to check the sensor performance. It can be seen that this type of topology improves the precise control of the resonance wavelength and therefore the sensor performance. But the enlargement of the structure and the tripling of the number of MHRRs would result in optical return and increased losses. As a final check, a network topology that combines all the horizontal, vertical and diagonal arrangements of MHRRs is designed. In this case, it can be seen that this type of array has improved the performance rather than several previous arrays. The advantage of this topology is the improvement in transmission to the output channel waveguide as well as the adjustment of the resonance wavelength, which is possible by removing multiple peaks. The reason for the improvement of this case could be attributed to the elimination of the return reflection wave due to each MHRR by the side MHRR. After the investigations, it can be concluded that double channel waveguides are much more practical in different presentations of MHRRs, because they make use of different topologies. It can also be seen that the use of only one MHRR is much more practical for optical sensor applications due to the better control of the light by the device, which prevents the return of light and precise adjustment of the resonance wavelength. Furthermore, the type of arrangement and combination of different MRR arrays to create functional topologies is determined according to the type of application and desired results.

**Table 2:** Design and study of various topology PC MHRR sensors using the one MHRR and waveguides to create an optimal and functional structure based on the proposed algorithm.

Case	Schematic design	Special description (⁕), feature (⁘) and drawbacks (⁜)	Design	Results			
			Rods (X × Z)	Simulation	λ (nm)	FWHM (nm)	TR (%)
**6**		**⁕** Design (1) + 1 Horizontal MRR **⁘** The FWHM has improved **⁘** High absorption	35 × 21		819.5	0.7	100
**7**		**⁕** Design (1) + Several horizontal MRR **⁘** Very wide FWHM **⁜** Large Footprint **⁜** Long simulation time **⁜** Need complex algorithms to optimize performance	35 × 21 to 43 × 21		819.5	5	85
**8**		**⁕** Design (2) + 1 Horizontal MRR **⁘** High transmission **⁘**FWHM has become narrower	35 × 21		817.7	1.1	97.4
**9**		**⁕** Design (2) + Several horizontal MRR **⁘** High transmission **⁘** Very wide FWHM **⁜** Large Footprint **⁜** Long simulation time **⁜** Need complex algorithms to optimize performance	35 × 21 to 43 × 21		818.8	2.4	98.4
**10**		**⁕** Design (3) + 1 Horizontal MRR **⁜**TR coefficient has become low	35 × 21		820	0.47	78
**11**		**⁕** Design (3) + Several horizontal MRR **⁘** High transmission **⁜** Large Footprint **⁜** Long simulation time **⁜** Need complex algorithms to optimize performance	35 × 21 to 43 × 21		820	0.6	100
**12**		**⁕** Design (3) + 1 Vertical MRR **⁘** High transmission **⁘**FWHM has become narrower **⁜** Multi-peak	27 × 29		818.9	0.25	96.6
**13**		**⁕** Design (3) + 1 Straight MRR **⁜** Multi-peak	33 × 25		815.8	0.63	96
**14**		**⁕** Design (3) + 1 Straight MR **⁘**FWHM has become narrower **⁜** Multi-peak	33 × 25		815.8	0.2	92.5
**15**		**⁕** Design (13) + (14) **⁘** Multi-peak has been deleted **⁜** TR coefficient has become low **⁜** Large Footprint **⁜** Need complex algorithms to optimize performance	39 × 25		821.7	0.35	60
**16**		**⁕** Design (13) + (14) + (15 or 12) + (12) + (13 or 14) **⁘** Multi-peak has been deleted **⁜** Large Footprint **⁜** Long simulation time **⁜** Need complex algorithms to optimize performance	39 × 29		818.2	1	96.2

**Figure 3:**
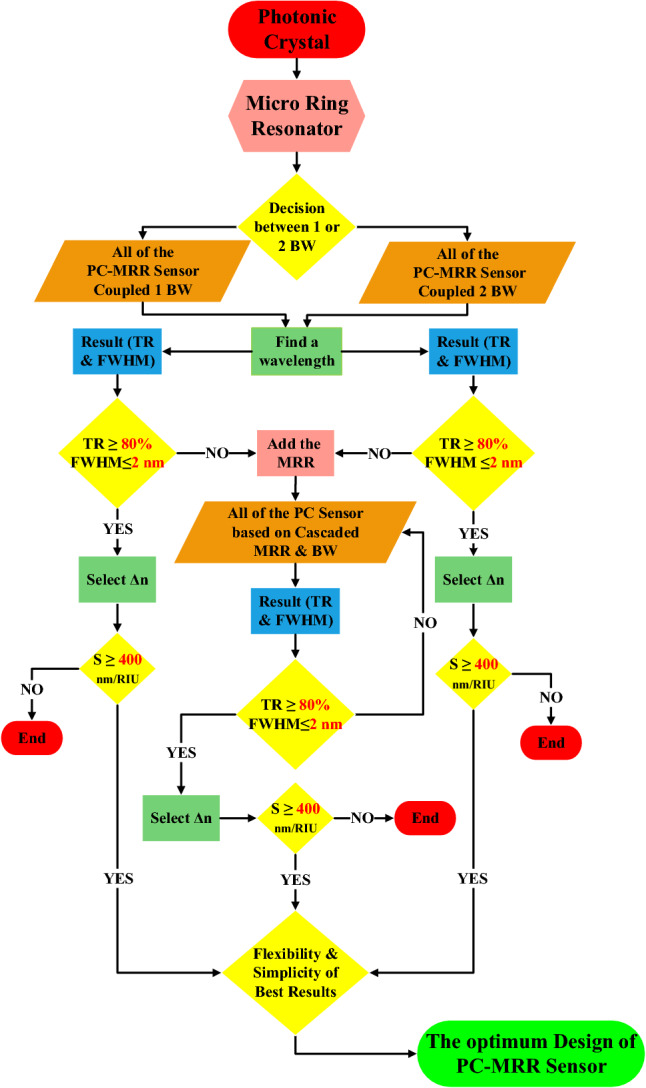
Process flow and representation of the sequential steps to finalize an optimum PC sensor mode.

After examining the topology of different optical sensors that were made using the proposed MHRR array, a final rule and algorithm can be presented in order to design and select the most optimal optical sensor structure. According to Fig. 3, the proposed process flow is based on the number of waveguides combined with MRR. By choosing the resonant wavelength, the investigation of the performance process of the proposed topologies begins. After determining the values of functional parameters and their suitability, the sensing performance of these topologies is measured. If these parameters are not met, the process of adding MRR and changes in their arrangement is implemented to improve the performance of the devices. These processes have high repeatability in order to achieve the most optimal design of optical sensors with regard to simplicity, flexibility, suitable results and theories in this field.

**Figure 4:**
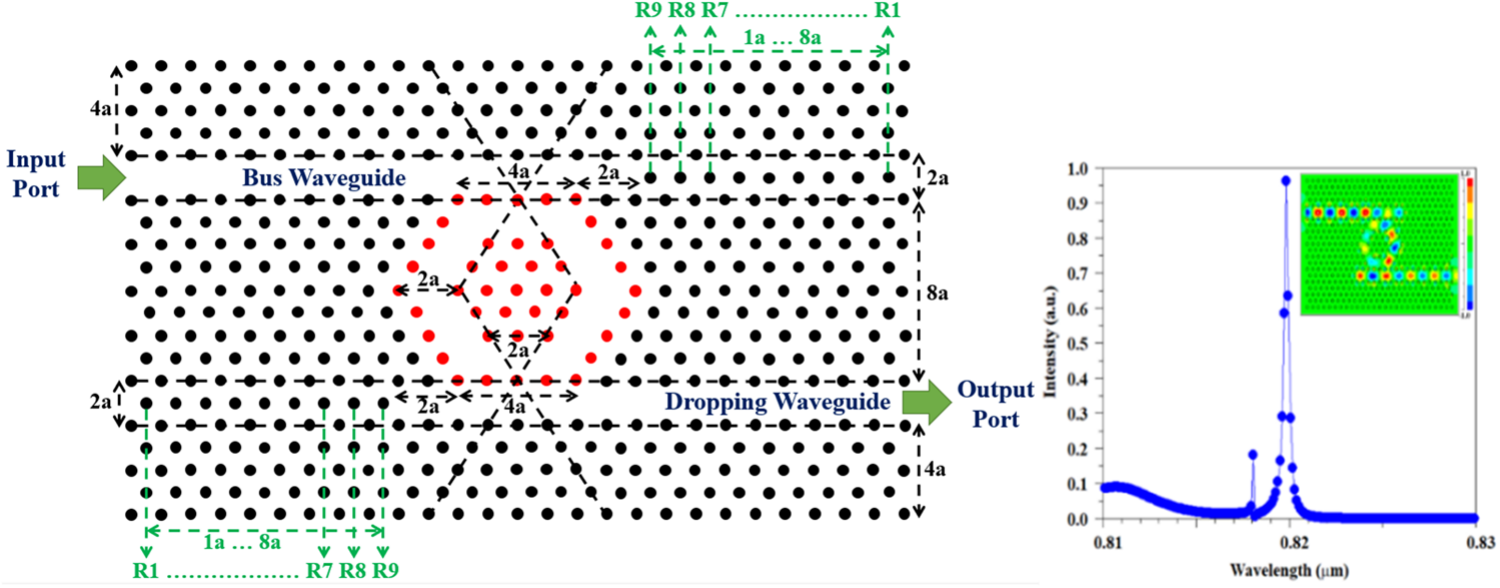
Schematic of optimized proposed PC sensor and 2D-FDTD intensity spectra and steady state electric field profile, which are composed of two channel waveguides coupled to MHRR.

**Figure 5:**
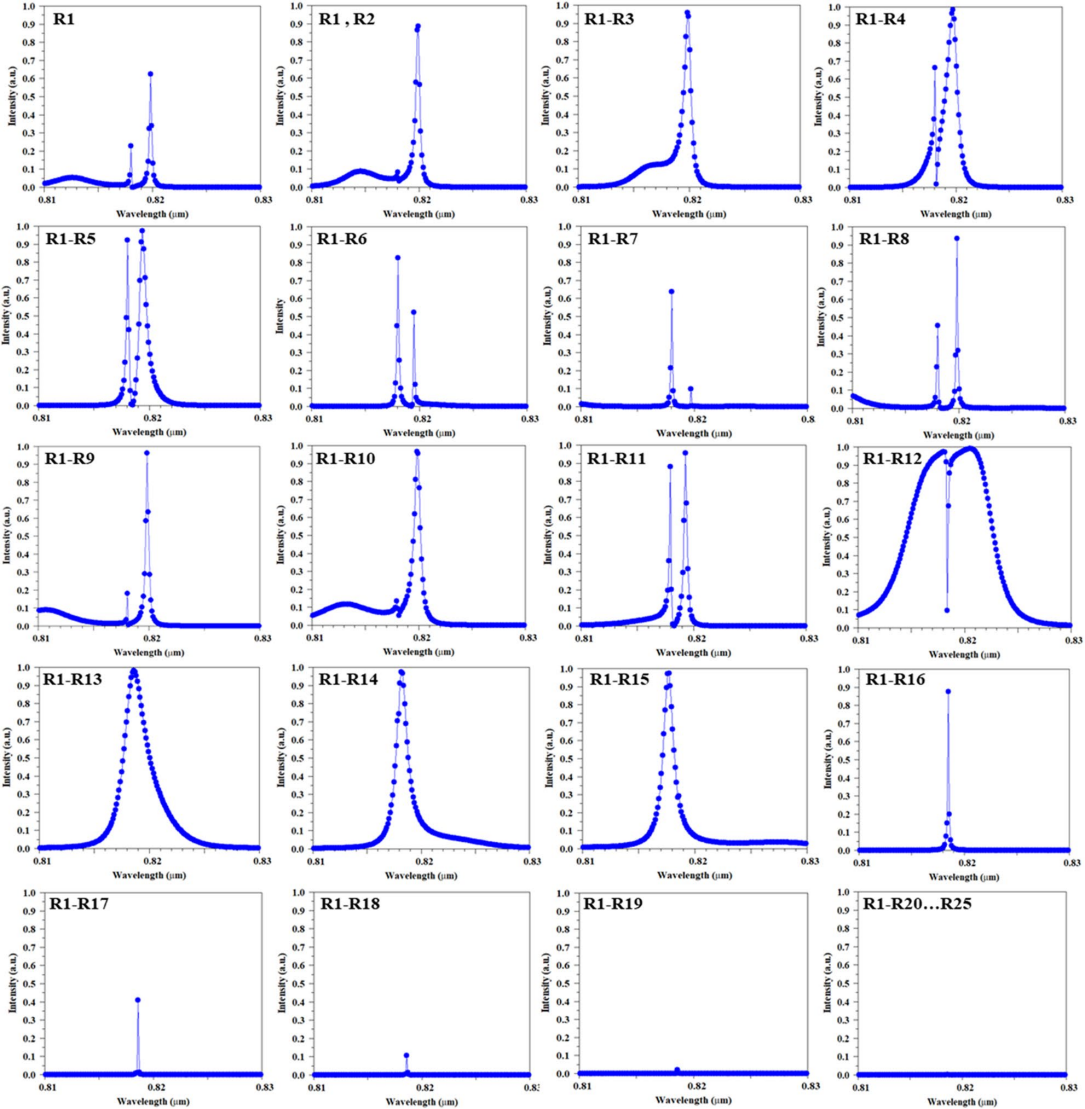
Investigation of the effects of reflector rods number on the end of waveguides on the sensor output in order to control the incoming and outgoing light for structure optimizing.

According to the proposed process flow and our results, the optical sensor with two channel waveguides has the best performance. As mentioned, this optical sensor prevents the back-radiation and return of light to the input waveguide due to its special design feature, which makes it perform well in terms of light separation and sensitivity and transmission for sensor applications. The final scheme of the proposed sensor and the result of its design are given in Fig. 4. It should be noted that this structure has its own complexity compared to other structures with a single channel waveguide, middle channel waveguide, horizontal channel waveguide and oblique channel waveguide. Considering that these structures are derived from add/drop filters, which naturally have 3 outputs along with one input. But in the case of sensors, only one output and one input are required. Therefore, it is necessary to remove or close two entries. In the sensor topology with a single channel waveguide or middle channel waveguide, this process can be easily done, because it is only possible by removing one of the waveguides. But in the sensor with more waveguides, the process of removing extra waveguides becomes a little more complicated and other design parameters are also involved. For this purpose, in the PC optical sensor, this process is done by adding dielectric rods to the other outputs that we intend to close, but the number of rods needed to place in these outputs may be challenging. For this purpose, in a comprehensive process, the results of adding dielectric rods step by step in the outputs have been investigated and are shown in Fig. 5. As can be seen from the results, by adding the first dielectric rod (R1), the transmission of the resonant wavelength shows a noticeable decrease. Meanwhile, by adding dielectric rods, we will see an increase in the transmission value, but adjusting the resonance wavelengths is difficult and disturbing peaks can be seen next to the resonance wavelength. But in the distance of 8 times of lattice constant and with the addition of R1–R9 rods, a good result has been obtained. After this step, by adding the dielectric rods, the resonant wavelength has moved towards broadening (R1–R15), and then, due to the closure of the optical waveguide, the transmission value finally tends to zero. The results indicate that the most optimal performance can be obtained when the number of reflector rods is equal to the number of rods in the proposed MHRR length or in other words, the size of reflector rods is equal to the length of the MHRR. In another way, it can be stated that the distance between the dielectric rods and the MHRR is 2a, which is equal to the width of the waveguide region. Finally, it can be concluded that the existence of similarity in quantity and sizes is important to achieve appropriate results.

**Figure 6:**
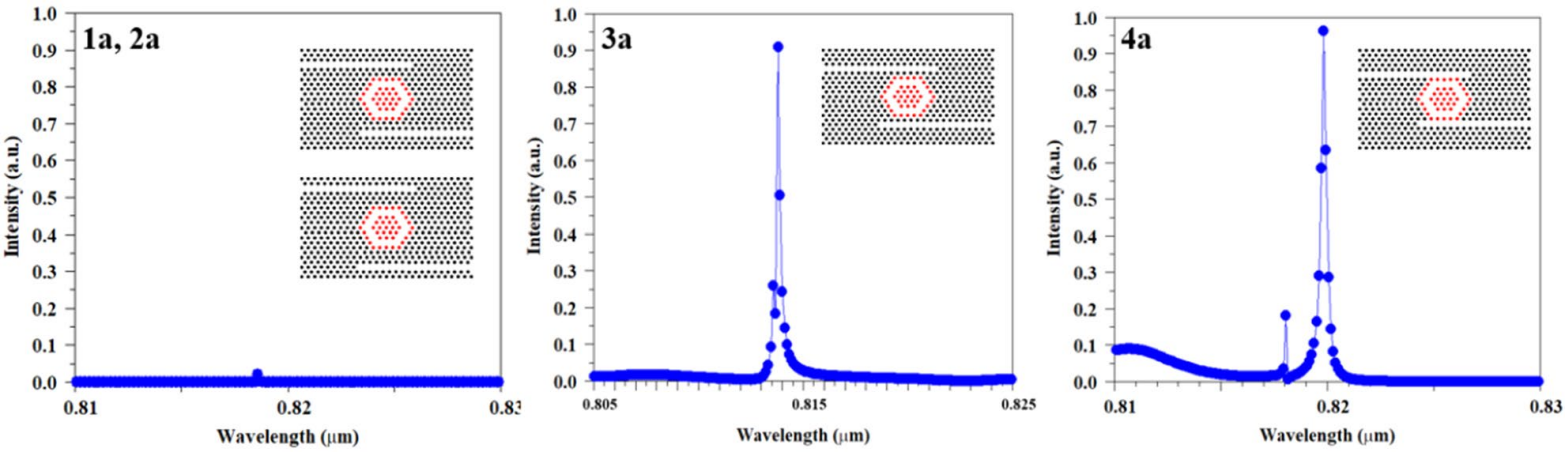
Investigating the effects of changing the distance between the waveguide and the ring resonator on the sensor output in order to optimize the proposed PC sensor.

Another parameter that should be checked is the coupling distance of MHRR with optical waveguides. The sensor output for different distance between 1 and 4a are given in Fig. 6. According to the obtained results, the shortest possible distance has shown the best performance due to the reduction of losses, stronger coupling and less reflection of light. The noteworthy point in this case is the equality of this distance with the width of the MHRR region, i.e., 4a, which is significant in its own way. Therefore, according to the results and investigations, it can be concluded that the existence of symmetry or similarity in the MRRs and waveguides geometries and their coupling could create an optimal design with suitable and acceptable results.

**Figure 7:**
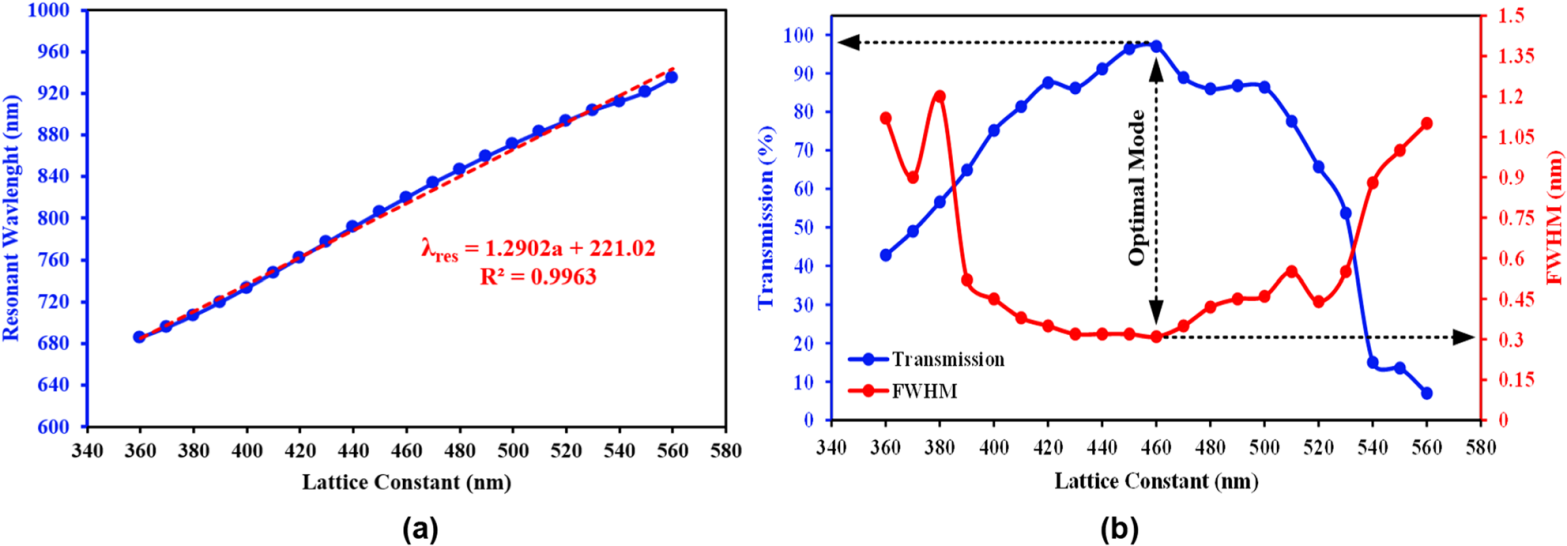
Changes in the lattice constant size and their effects on, (**a**) resonant wavelength, (**b**) FWHM and transmission in order to obtain the optimized radius of the proposed sensor.

**Figure 8:**
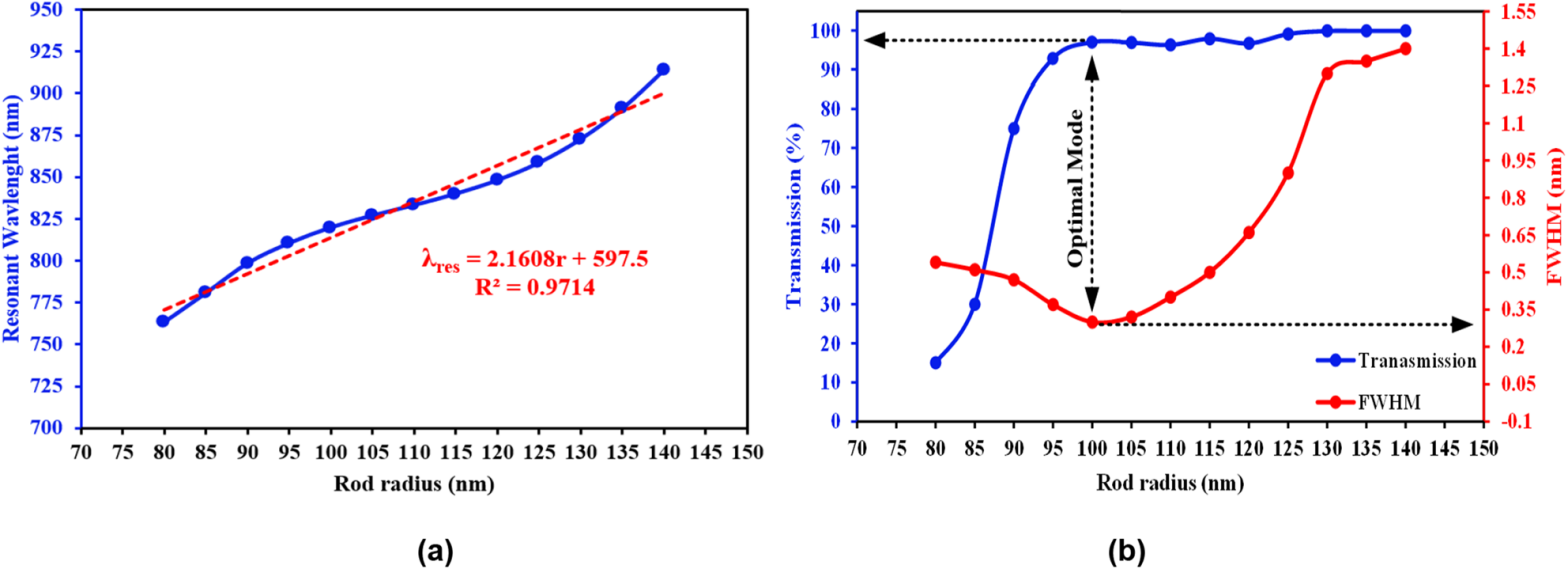
Changes in the dielectric rods radius and their effects on, (**a**) resonant wavelength, (**b**) FWHM and transmission in order to obtain the optimized radius of the proposed sensor.

In the optimization, it is very important to check the values of the structural parameters such as the lattice constant and the radius of the dielectric rods, as well as their effects on the results. For this purpose, in the design optimization, these parameters have been examined on the transmission coefficient as an important factor in the absence of loss in optical sensors, as well as FWHM, which determines other sensor parameters. According to Fig. 7, by increasing the lattice constant from 340 nm, it has an upward trend and the FWHM has a decreasing trend, and at the value of 460 nm, it has reached its optimal mode, the highest value of transmission and the lowest FWHM, and then it has a reverse trend. Figure 7a also indicates a quasi-linear trend for the resonant wavelength changes versus lattice constant, which indicates the logical and approximately linear performance of the structure in relation to the sensor parameters changes. The results of the changes in the radius of the dielectric rods are shown in Fig. 8. This figure reveals that by increasing the radius of the dielectric rods, the transmission has an upward trend and the FWHM has narrowed. From the value of 100 nm onwards, the transmission has been almost a constant value, while the FWHM has had its narrowest value, which shows that 100nm is the optimal radius for the dielectric rods. A quasi-linear trend for resonance wavelength versus rods radius is also evident in Fig. 8, which indicates the proper design of the structure for the desired applications.

## Application as gas sensor

**Figure 9:**
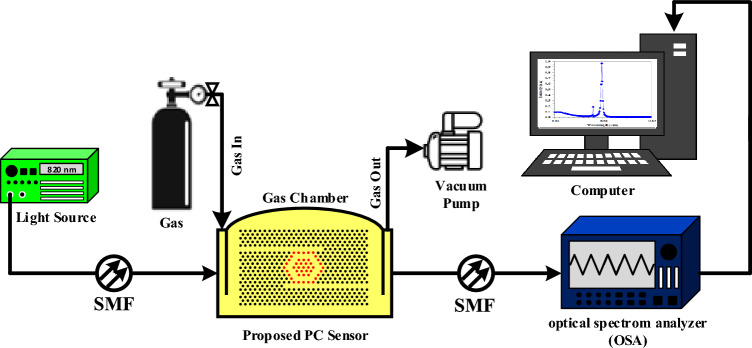
Proposed experimental setup of the proposed gas sensor using 2D PC structure.

In this section the performance of the proposed optical sensor for the detection of pure gases such as air, O_2_, C_2_H_2_, C_2_H_4_ and C_2_H_6_ has been analyzed. The most frequently used fabrication methods for 2D-PC nano-scale structures include direct-writing lithography, deep etching, self-assembly, nano-imprint (NIL), interference and electron-beam lithography (EBL). Among them the NIL process can be fabricated on a silicon wafer using the conventional EBL process. Also, EBL can be created by using a JBX-9300FS system (JEOL Ltd.) with a negative CAR-type resist of NEB-22S68 (Sumitomo Ltd.)^44^. 2D PC sensors that are designed and manufactured in the air substrate have a good performance and simplicity for use in oil and gas industries^45^. In this type of optical sensor, the desired gases are easily replaced by the medium (air) of dielectric rods or air cavities inside the PC, therefore its refractive index is changed and can be detected^33^. The proposed device works as a selective sensor for detecting different gases, which determines its type according to each specific intensity peak for each gas. Also, by checking each peak with the reference peak, other parameters such as sensitivity can be determined. According to Fig. 9, in order to provide the laboratory setup scheme of the proposed optical sensor, a laser is used as a light source to send a pulse and a single-mode fiber (SMF) is used to couple the incoming light to the optical sensor, which is finally etched into the device^46^. The light output from the optical sensor, which carries information from the interaction of light and analyte, is sent to the optical spectrum analyzer (OSA) and then the output spectrum is displayed on the monitor. In order to create an isolated environment for injecting the desired gases, a chamber could be used, which has an inlet for gas injection and an outlet for vacuuming.

**Figure 10:**
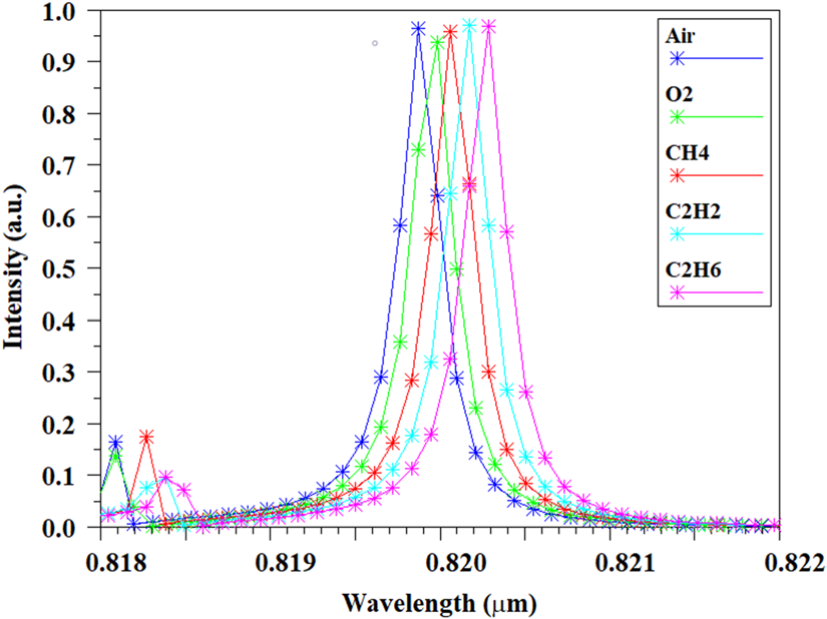
The position of the resonance peak as a function of the refractive index for; Air, O_2_, CH_4_, C_2_H_2_ and C_2_H_6_ sensor devices.

Air is used as a reference for sensing other pure gases. The replacement of O_2_, C_2_H_2_, C_2_H_4_ and C_2_H_6_ gases by air has been equated and finally simulated, which has shown itself as a resonance wavelength according to Fig. 10. In the investigated gases, as their mass becomes heavier, the refractive index increases in the same proportion as compared to air. According to Table 3, the results of the simulations carried out by the 2D-FDTD method for the gas sensing topics under investigation indicate the FWHM is about 0.31 nm, and the TR is above 96%. Another important parameter that is highly emphasized in optical sensors is sensitivity (S), which shows the interaction of the light and analyte in the sensor structure. Sensitivity in optical sensors is defined as follows^33^:

1 $$ S = \frac{\Delta \lambda }{{\Delta n}} $$

where ∆λ is the displacement of the resonance wavelength and Δ*n* is the change of the gas refractive index due to the placement of the analyte in the structure of the optical sensor. The sensitivity for proposed gas sensor is obtained above 862 nm/RIU.

**Table 3:** The values of resonance peak, FWHM, TR, QF, sensitivity and FOM; Air, O_2_, CH_4_, C_2_H_2_, C_2_H_6_ sensor devices.

Samples	Reflective Index	λ (nm)	FWHM (nm)	TR (%)	QF	Sensitivity (nm/RIU)	FOM
Air	1.000273	819.87	0.31	96.6	2664.7	Ref	Ref
Oxygen (o_2_)	1.0002916	819.99	0.31	96	2645.1	6451	2080
Methane (CH_4_)	1.00044023	820.06	0.312	96	2628.4	1136	3641
Acetylene (C_2_H_2_)	1.00055994	820.17	0.311	97.3	2637.2	1045	3360
Ethane (C_2_H_6_)	1.00074873	820.28	0.312	97	2629.1	862	2762

The existence of a high-QF is desirable to increase the detection limit of resonance peaks. The QF is obtained as shown in the follow^1^:

2 $$ QF = \frac{Resonant\;Wevelength}{{FWHM}} $$

For this sensor, a QF exceeding 2620 has been achieved.

The FOM parameter is used to determine the accuracy and precision of the biosensor. The FOM for the proposed sensor is about 2960 RIU^−1^. This parameter can be expressed by the following relation^45^:

3 $$ FOM = \frac{S}{FWHM}. $$

The results of the design and simulation of the proposed optimized sensor show its proper performance in order to be used in OICs and competitive with other devices in this field. Table 4 shows a comparison of the research in this field. The classification and complete review of the possible cases in the design of optical sensors based on PC MRR have led to the emergence of a novel algorithm to optimize these optical devices with high efficiencies. The results of this algorithm are clear in the comparison between the results obtained from the proposed optimized optical sensor with other sensors. The attention to the process of arranging optical waveguides with MRR and creating different topologies in these optical devices is important because the existence of diversity in these structural arrays causes different results with different applications. In the majority of researches, the investigation of structural parameters has been the only proposed solution to create a structure with optimal performance. Meanwhile, by examining various topologies, creating the optimal case in these structures can be brought to an earlier step, that is, structure design. In this process, the design of the structure can be selected according to the type of its application, and then the parameter optimization process can be performed which causes better performance. Also, examining the parameters that are less paid attention to them in these structures, such as the reflector rods and the coupling area, can have different effects on the performance of the structure, which is considered very necessary. In this research, an attempt has been made to draw the way forward for the work areas of this field in a selective process, so that the need for correct and error processes can be minimized.

**Table 4:** Comparison of the numeric values for the sensitivity, FOM, TR, QF and footprint of the proposed design with previous designs for evaluating the performance of the proposed sensor.

References	Sensor configuration	Sensitivity (nm/RIU)	FOM (RIU^−1^)	TR (%)	QF	Footprint (µm^2^)
^47^	1D-PC ring resonator	248	–	–	1200	–
^48^	Optimized ring resonator	229	–	–	1339	–
^49^	Plasmonic ring resonator	636	211	–	269	–
^1^	Optimized cascade micro-ring resonator	1024	366	–	524	288
^50^	WGM ring resonator	361	–	–	1143	–
^36^	MIM square ring resonator	1320	16.7	–	18	–
^33^	Micro-ring resonator	745	593	97	1092	123
^34^	Topological ring resonator	798	725	–	1256	–
^35^	Hybrid split-ring resonator	1250	2083	–	2412	–
This work	Optimized PC micro-ring resonator	860 < S < 6500	2960	96.5	2640	113

## Conclusion

In this paper, in comprehensive research and step-by-step process, using symmetric MHRR based on PCs, various topologies that can be achieved for the application of optical sensors have been attempted. Different arrays of waveguide and MHRRs next to each other have created different geometries. The output spectrum results from the simulation of these topologies are matched with the theoretical analysis. Examining different configurations has provided us a suitable process flow in order to realize the best design of optical sensor with the lowest cost in terms of money and time. After various investigations of all topologies, it can be concluded that, the use of only one MHRR combined with double channel waveguides is much more practical for optical sensor applications due to the better control of the light by the device which prevents the return of light. It also has the merit of precise adjustment of the resonance wavelength. Finally, based on the results and the proposed algorithm, the optimal sensor has been selected and the effects of the reflector rods and the radius of the dielectric rods and the lattice constant have also been investigated. The existence of symmetry, balance and similarity in the designs has been an important and fundamental factor in obtaining the most optimal results. In order to check the performance of the proposed optimal topology in terms of sensor, it has been used to detect O_2_, C_2_H_2_, C_2_H_4_ and C_2_H_6_ gases. The results indicate that the TR is equal to 96%, the FWHM is about 0.31 nm, the QF is about 2640, the S is above of 860 nm/RIU, and the FOM is about 2960, which is competitive with other devices in this field. The step-by-step and logical process of this article in order to design and achieve optimal sensors and observe the effects of arrays and different topologies of sensors based on PC MRRs can be of great help to researchers in this field.

## Data Availability

The datasets used and/or analyzed during the current study available from the corresponding author on reasonable request.
